# Microscopic Unilateral Approach for Bilateral Decompression of Lumbar Spinal Stenosis

**DOI:** 10.34172/aim.2022.117

**Published:** 2022-11-01

**Authors:** Hülagü Kaptan, Ömür Kasimcan, Şafak Özyörük, Murat Yılmaz

**Affiliations:** ^1^DokuzEylül University, Medical School, Department of Neurosurgery, Izmir, Turkey; ^2^Istinye University, Medical School, Department of Neurosurgery, Istanbul, Turkey

**Keywords:** Bilateral decompression, Lumbar spinal stenosis, Microscope, Unilateral approach

## Abstract

**Background:**

This is a study based on single-surgeon data on spinal stenosis surgery via microscopic approach. The aim is to evaluate the effectiveness of the unilateral approach to bilateral decompression and the usage of Taylor retractors and brain spatula in patients with spinal stenosis.

**Methods:**

This is a retrospective study on bilateral decompression for lumbar spinal stenosis using a microscopic unilateral approach by a single surgeon, between April 2015 and March 2018. In total, 50 patients were operated due to single level lumbar spinal stenosis. All patients were evaluated by preoperative and postoperative plain radiographs and magnetic resonance (MR) images. Walking distance (WD), visual analog scale (VAS) for pain and Odom’s criteria were evaluated for follow-up.

**Results:**

One level of the lumbar spine was surgically decompressed in all patients. The median age of patients was 64.6 (51– 82). Of the patients, 72% (36) were women, and 28% (14) were men. Most patients had refractory low back pain (96%) after conservative treatment. The stenotic levels of the cases were as follows: L3–4, 23(46%); L4–5, 24(48%); and L5–S1, 3 (6%). VAS scores decreased in all patients after surgery. According to Odom’s criteria, an excellent or good score was found in 43 patients at the 12th follow-up examination. WDs increased up to 1000 meters for 41 patients.

**Conclusion:**

The microscopic unilateral approach to bilateral decompression is an effective method for decompression in spinal stenosis. Via this approach, surgical trauma is reduced and surgically induced instability is avoided as much as possible.

## Introduction

 A complicated process that can involve degenerative disc, arthropathy of facet joints, hypertrophy of flavum ligament, spondylosis, and occasionally spondylolisthesis, stenosis at the intervertebral junctions develops focally. The main symptoms are low back pain, radiculopathy, and numbness exacerbated by walking.^1^ Surgical decompression is reported to be an effective treatment, even in the elderly. Protection of the stability of the spinal zone is important for this operation.^1^

 Wide laminectomy and undercutting of the partial or complete facetectomy with foraminotomy are conventional treatments of spinal stenosis. Less extensive resection is recommended to maintain normal spinal anatomy.^2-9^

 Deformity or instability of lumbar spinal disorders used to be treated via fusion operations. However, concerns about the long-term effects of fusion on neighboring segments resulted in the development of dynamic stabilization.^10,11^

 To lower the risk of postoperative spondylolisthesis, limited resection can be performed. The primary treatment for lumbar stenosis includes bilateral spinal canal decompression with unilateral laminotomy.^12-16^

 Many different techniques for decompression have been described for spinal stenosis. Bilateral decompression through a unilateral approach can be done effectively and sufficiently. Surgery using a unilateral approach preserves contralateral facet joints and nerve structures, thus limiting postoperative instability.^17,18^

 The aim of the present study is to evaluate the effectiveness of the unilateral approach to bilateral decompression and the usage of Taylor retractors and brain spatula in spinal stenosis.

## Materials and Methods

 A total of 50 patients with spinal stenosis were included in this study; these patients underwent surgery at two centers (Dokuz Eylül University, Faculty of Medicine, Department of Neurosurgery, İzmir, Turkey and Selcuk University, Faculty of Medicine, Department of Neurosurgery, Konya, Turkey) between April 2015 and March 2018. The approval of the local institutional review board was received with the number of 2017/319 from the ethics committee of our institution. All the patients were operated on viabilateral decompression using the microscopic unilateral approach.

 This retrospective study was performed using data derived from 50 selected patients with spinal stenosis refractory to conservative treatment for at least six months who underwent bilateral decompression: unilateral laminectomy for bilateral decompression (ULBD) using an operating microscope. Assessments of the patients’ neurologic status were made by neurological examination and pre- and postoperative radiological investigations. Inclusion criteria for this study were (1) neurogenic claudication and/or chronic nerve root compression findings, (2) radiologically confirmed spinal stenosis, (3) receiving a minimum of 3 months of conservative treatment, and (4) single-level spinal stenosis. Patients with lumbar disc herniation, previous spinal surgery, spinal trauma, spinal infection, spinal tumor and multilevel spinal stenosis or spondylolisthesis, vascular claudication, and hip or knee osteoarthritis were excluded from the study.

 Spinal canal decompression with this technique can be applied in selected cases. In other words, there is no need to be a reference center for the application of this technique.

 A single surgeon using a microscopic approach in their surgery performed the operation on a series of selected patients with spinal stenosis. Patients were evaluated by radiography and magnetic resonance imaging (MRI).

**Figure 1:**
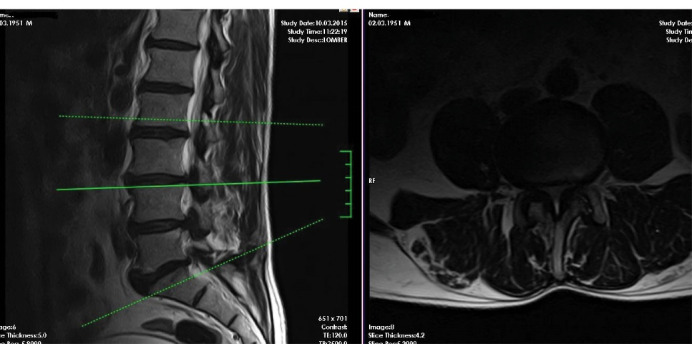
Preoperative Magnetic Resonance Images. Sagittal and axial T2 view for L3-4 level spinal stenosis.

**Figure 2:**
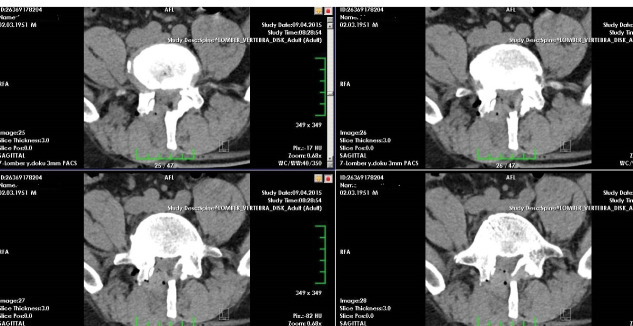
Postoperative Computerized Tomography Scans in Axial Section to Check Operative Levels.

 Preoperative radiologic examination included roentgenogram, MRI, and computerized tomography (CT)for surgical decisions for all patients (Figure 1). We performed regular follow-up examinations atone, six, and twelve months postoperatively. We checked operative levels with CT scan for all on the first postoperative day for decompression size (Figure 2). We also viewed the stabilization with dynamic lumbar x-rays at the final follow-up examinations. The visual analog pain scale (VAS), the walking distance (WD) scale, and Odom’s criteria (Table 1) were determined at the pretreatment, the third-month postoperative, and the 12th-month postoperative examinations.

**Table 1:** Rating Protocol of Odom’s Criteria

**Rate**	**Description**	**Criteria**
1	Excellent	Completely relieved of symptoms, and daily lives and occupations not impaired.
2	Good	Intermittent discomfort, but no interference in occupational activities.
3	Fair	Subjective improvement, but physical activities still significantly limited.
4	Poor	No improvement, or symptoms have deteriorated.

###  Statistical Analysis

 Descriptive statistics (mean, median) and non-parametric analyses were used for statistical analysis. Statistical analyses were performed using SPSS 16 (IBM, USA). McNemar’s test was used for the pre- and postoperative comparison of categorical data, and the Wilcoxon test was used for the pre- and postoperative comparison of the quantitative data (VAS score, Odom’s criteria). Statistical significance was set at *P* < 0.05.

###  Surgical Procedure

**Figure 3:**
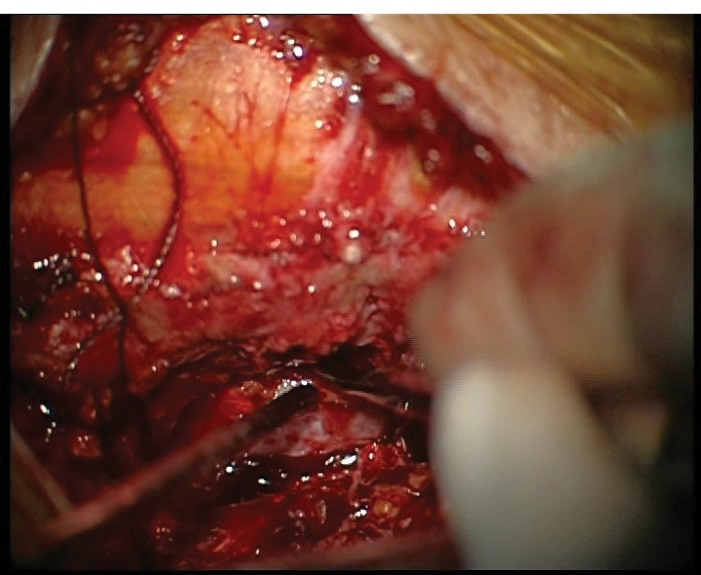
Perioperative Image Demonstrating Exposure During the Surgical Procedure. Showing contralateral decompression limits with dissector.

 Patients were set and positioned for a hemi partial-laminectomy. The level of interest was localized with a perioperative C-arm and the operating microscope was placed to the surgical field. Surgery was directed for nerve decompression and partial resection of the facet for complete removal of the ligamentum flavum to maintain a stable and balanced spine. Spinous processes, the interspinous ligaments and the integrity of the physiological muscular attachment of the opposite side were preserved via microsurgical approach. The retraction was made by Taylor retractors to reach the surgical site. The spinal cord was protected with a brain spatula during contralateral decompression (Figure 3).

## Results

 The median age of patients was 64.6 (51–82). Of the patients, 72% (36) were women, and 28% (14) were men (Table 2). The symptoms duration was 57 months, with a mean of 25 months.

**Table 2:** Clinical and Demographic Data of Patients

**Parameters **	**n**	**%**
Total number of patients	50	100
Gender		
Male	14	28
Female	36	72
Stenotic level of the lesion		
L3–4	23	46
L4–5	24	48
L5–S1	3	6
Complaints		
Radiculopathy	42	84
Low back pain	48	96
Neurologic claudication	45	90
Sensory abnormalities	26	52
Motor abnormalities	18	36
Age (y)	64.6 (51–82)	

 A single level of the lumbar was surgically decompressed for stenosis. Discectomy was performed in 13 (26%) patients with concomitant disc herniation. There were no neurological injuries, dural tears, or bleeding. No infections or other complications were reported in the follow-up period. There was no stenosis at the operation level after ULBD. No revision or secondary fusion was required in the follow-up period for any of the patients.

 Most patients had refractory low back pain (96%) after conservative treatment. They also experienced radiculopathy (84%), neurological claudication (90%), motor abnormalities (36%), and sensorial abnormalities (52%) at the same time. The stenotic levels of the cases were as follows: L3–4, 23 (46%); L4–5, 24 (48%); and L5–S1, 3 (6%) (Table 2). The mean operation time was 93.4 minutes.

 Mean VAS scores were 8.61 pretreatment and 1.44 at the last follow-up examinations (*P* < 0.001) (Table 3). Pretreatment WD was 0–100 meters in 39 patients and post treatment WD was up to 1000 meters in 41 patients (*P* < 0.001). Odom’s criteria scores were poor in 42 patients before surgery and excellent and good in 43 patients at the last follow-up examinations (Table 3).

**Table 3:** Odom’s Criteria and VAS Scores Preoperative and at 12 Months Postoperatively

**Description**	**Preoperative**	**12-month follow-up**	***P*** ** value**
VAS score	8.61 ± 2.34	1.44 ± 1.65	< 0.001
Odom’s criteria			
Excellent	0	19 (38%)	< 0.001
Good	0	24 (48%)	< 0.001
Moderate	8 (16%)	7 (14%)	> 0.05
Poor	42 (84%)	0	< 0.001

VAS,Visual Analog Scale for Pain.

 We evaluated instability with dynamic lumbar X-rays in all patients at the final follow-up examinations. There was no up-to grade 1 listhesis in any patients, but we found a grade 1 ( < 10%) in two patients (4%). They did not have radicular pain, neurological claudication, or motor and sensory deficits. Odom’s criteria scores were good or excellent in 36 patients, but only poor or moderate in 9 patients with lumbar spinal stenosis (LSS).

## Discussion

 In recent years, minimally invasive procedures have been used more frequently in spinal surgeries. Before this, total laminectomy was performed and the patients benefited from it, but authors have varying results for the use of laminectomy at the minimal invasive procedures. As reported by Aryanpur and Ducker, complete decompression may not be necessary for symptomatic relief.^2^ However, Thomas et alreported that the degree of decompression after laminotomy or laminectomy did not reflect statistically in clinical outcomes.^7^

 Minimally invasive interventions instead of total laminectomy have been widespread in the management of spinal stenosis over time to prevent lumbar instability.^16,19-23^ Numerous surgical methods for lumbar spine decompression have been described in recent years. We used a unilateral access for bilateral decompression in our cases, and VAS and Odom’s scores were found to be statistically significant for ULBD.

 In general, it has been shown that durotomy rates for laminectomy vary between 5 and 15%. The rate of dural tears was 2–9% in bilateral laminotomy and 3.5–12%. in ULBD.^3,16,22,24^ We did not encounter other complications in our cases, especially dural tears.

 A study by Turner et al showed that in 64% of patients who underwent surgery for lumbar spinal stenosis, good to excellent results were achieved.^25^ Similarly, the clinical case series by Aryanpur and Ducker. reported good results in 90% (29 of 32 patients) with decompressive laminotomies and foraminotomies at appropriate levels.^2^ Kaptan et al reported that short-term partial recovery was observed in 62.5% of cases with total laminectomy.^1^

 Recently, less invasive and more restricted interlaminar decompression by bilateral laminotomies and foraminotomies have been applied because this surgical approach preserves neural structures and the trauma effect of the surgical approach is minimized. Another advantage of this method is that, in the treatment of multiple segmental stenosis, the deterioration of the natural anatomy is prevented by preserving the spinous processes. The loss of these ligaments poses a great risk for spinal instability. In addition, the central line structures (interspinous ligaments and dorsolumbar fascia), together with the spinous process, are of great importance for the natural anatomic structure.^3,6,26,27^

 However, a comparison of laminotomy and laminectomy indicated no differences between these twomethods.^7,27,28^ This finding suggests that hemilaminectomy does not only provide good surgical outcomes but also provides surgical convenience and safety. ^3,7,22,24,28,29^ Yet, our own experience led to different results. Our results with ULBD were better, and Odom’s criteria scores were good and excellent in 43 patients (86%).

 Because it is less invasive, ULBD for spinal stenosis yields better results and does not disrupt the natural anatomy. The main purpose of the surgery is to relieve the patient with adequate decompression. In this way, instability is prevented, and lumbar fusion surgery is not required.

 Open decompression in lumbar spinal stenosis can yield good to excellent results in 60–65% of patients. However, wide-scale deterioration of bone and muscle structures can lead to instability of flexion. If there is a large dead space, it is obvious that this will be the ideal environment for colonization or scar formation. These complications can lead to chronic pain and failed back surgery syndrome. For this reason, and so as not to compromise nerve decompression over time, there is a tendency toward minimally invasive surgery.^1,16,17,30-32^

 ULBD was reported to be as effective as open decompression in improving function (Oswestry Disability Index score) according to a prospective clinical study by Mobbs et alon 79 patients comparing ULBD and a standard open laminectomy.^33^ In addition, shorter hospitalization time, shorter mobilization time, and reduced postoperative use of opioids are additional benefits of the ULBD procedure. ULBD is reported to provide no instability effect and improved postoperative patient comfort. Also, Ho et al evaluated the biomechanical stability of porcine lumbar spines using a standardized motion tracking system following standard open laminectomy and unilateral as well as bilateral laminectomy.^34^ Instability was increased in the open laminectomy group compared to the unilateral and bilateral laminotomy groups. Theoretically, the decompression of relieves the narrowing spinal canal and neural canal extension and, thus, reduces the symptoms.^10,33,34^ This is in parallel to our follow-up results.

 Ozer et al reported loosening screws and breakage after lumbar single-level dynamic stabilization in their study.^35^ They also remarked that revision surgery was undertaken for all patients with screw loosening or breakage.

 Posterior fusion with transpedicular screws carries the risk of denervation of paraspinal muscles. Cha et al noted denervated erector spinae muscles at 12 months following pedicle screw fixation with fusion.^36^ This is closely related to postoperative long-term low back pain and in stabilization. Problems found in another study included nerve root or vascular injury due to instrumentation-related complications.^37^ In our series, the ULBD technique seemed not to cause any complications. Some studies report favorable outcomes with ULBD, but there is still discussion about which technique is best for decompression of the lumbar spinal stenosis. In parallel with previous publications, we believe that minimally invasive techniques will be more popular in the future.^38-40^ Furthermore, we suggest that the use of Taylor retractors and protection of spinal cord using brain spatula may facilitate the procedure.

 The main limitations of this study include lack of a control group, its retrospective design, and data confined to the experience of a single center. Moreover, our analysis did not include perioperative and clinical data such as duration of hospitalization, operative time, and blood loss. Thus, interpretation of our results and extrapolation to larger populations must be made cautiously.

 To conclude, our study shows the feasibility of applying the minimally invasive unilateral bilateral decompression approach for single-level spinal stenosis in selected cases.

## References

[1] Kaptan H, Kasimcan O, Cakiroglu K, Ilhan MN, Kilic C (2007). Lumbar spinal stenosis in elderly patients. Ann N Y Acad Sci.

[2] Aryanpur J, Ducker T (1990). Multilevel lumbar laminotomies: an alternative to laminectomy in the treatment of lumbar stenosis. Neurosurgery.

[3] diPierro CG, Helm GA, Shaffrey CI, Chadduck JB, Henson SL, Malik JM (1996). Treatment of lumbar spinal stenosis by extensive unilateral decompression and contralateral autologous bone fusion: operative technique and results. J Neurosurg.

[4] Jane JA Sr, Jane JA Jr, Helm GA, Kallmes DF, Shaffrey CI, Chadduck JB (1996). Acquired lumbar spinal stenosis. Clin Neurosurg.

[5] Nakai O, Ookawa A, Yamaura I (1991). Long-term roentgenographic and functional changes in patients who were treated with wide fenestration for central lumbar stenosis. J Bone Joint Surg Am.

[6] Poletti CE (1995). Central lumbar stenosis caused by ligamentum flavum: unilateral laminotomy for bilateral ligamentectomy: preliminary report of two cases. Neurosurgery.

[7] Thomas NW, Rea GL, Pikul BK, Mervis LJ, Irsik R, McGregor JM (1997). Quantitative outcome and radiographic comparisons between laminectomy and laminotomy in the treatment of acquired lumbar stenosis. Neurosurgery.

[8] Young S, Veerapen R, O’Laoire SA (1988). Relief of lumbar canal stenosis using multilevel subarticular fenestrations as an alternative to wide laminectomy: preliminary report. Neurosurgery.

[9] Pao JL, Lin SM, Chen WC, Chang CH (2020). Unilateral biportal endoscopic decompression for degenerative lumbar canal stenosis. J Spine Surg.

[10] Kaptan H, Kasimcan O, Birler S, Ayaz M (2011). Early results of non-fusion dynamic stabilization with InterS. Minerva Ortop Traumatol.

[11] Weber C, Lønne G, Rao V, Jakola AS, Solheim O, Nerland U (2017). Surgical management of lumbar spinal stenosis: a survey among Norwegian spine surgeons. Acta Neurochir (Wien).

[12] Choi WS, Oh CH, Ji GY, Shin SC, Lee JB, Park DH (2014). Spinal canal morphology and clinical outcomes of microsurgical bilateral decompression via a unilateral approach for lumbar spinal canal stenosis. Eur Spine J.

[13] Deschuyffeleer S, Leijssen P, Bellemans J (2012). Unilateral laminotomy with bilateral decompression for lumbar spinal stenosis: short-term risks in elderly individuals. Acta Orthop Belg.

[14] Jang JW, Park JH, Hyun SJ, Rhim SC (2016). Clinical outcomes and radiologic changes after microsurgical bilateral decompression by a unilateral approach in patients with lumbar spinal stenosis and grade I degenerative spondylolisthesis with a minimum 3-year follow-up. Clin Spine Surg.

[15] Liu X, Yuan S, Tian Y (2013). Modified unilateral laminotomy for bilateral decompression for lumbar spinal stenosis: technical note. Spine (Phila Pa 1976).

[16] Palmer S, Turner R, Palmer R (2002). Bilateral decompression of lumbar spinal stenosis involving a unilateral approach with microscope and tubular retractor system. J Neurosurg.

[17] Mariconda M, Fava R, Gatto A, Longo C, Milano C (2002). Unilateral laminectomy for bilateral decompression of lumbar spinal stenosis: a prospective comparative study with conservatively treated patients. J Spinal Disord Tech.

[18] Adachi K, Futami T, Ebihara A, Yamaya T, Kasai N, Nakazawa T (2003). Spinal canal enlargement procedure by restorative laminoplasty for the treatment of lumbar canal stenosis. Spine J.

[19] Haba K, Ikeda M, Soma M, Yamashima T (2005). Bilateral decompression of multilevel lumbar spinal stenosis through a unilateral approach. J Clin Neurosci.

[20] Kuo CC, Merchant M, Kardile MP, Yacob A, Majid K, Bains RS (2019). In degenerative spondylolisthesis, unilateral laminotomy for bilateral decompression leads to less reoperations at 5 years when compared to posterior decompression with instrumented fusion: a propensity-matched retrospective analysis. Spine (Phila Pa 1976).

[21] Mackay DC, Wheelwright EF (1998). Unilateral fenestration in the treatment of lumbar spinal stenosis. Br J Neurosurg.

[22] Thomé C, Zevgaridis D, Leheta O, Bäzner H, Pöckler-Schöniger C, Wöhrle J (2005). Outcome after less-invasive decompression of lumbar spinal stenosis: a randomized comparison of unilateral laminotomy, bilateral laminotomy, and laminectomy. J Neurosurg Spine.

[23] Weiner BK, Walker M, Brower RS, McCulloch JA (1999). Microdecompression for lumbar spinal canal stenosis. Spine (Phila Pa 1976).

[24] Spetzger U, Bertalanffy H, Reinges MH, Gilsbach JM (1997). Unilateral laminotomy for bilateral decompression of lumbar spinal stenosis. Part II: clinical experiences. Acta Neurochir (Wien).

[25] Turner JA, Ersek M, Herron L, Deyo R (1992). Surgery for lumbar spinal stenosis Attempted meta-analysis of the literature. Spine (Phila Pa 1976).

[26] Yang F, Chen R, Gu D, Ye Q, Liu W, Qi J (2020). Clinical comparison of full-endoscopic and microscopic unilateral laminotomy for bilateral decompression in the treatment of elderly lumbar spinal stenosis: a retrospective study with 12-month follow-up. J Pain Res.

[27] Tuite GF, Stern JD, Doran SE, Papadopoulos SM, McGillicuddy JE, Oyedijo DI (1994). Outcome after laminectomy for lumbar spinal stenosis. Part I: clinical correlations. J Neurosurg.

[28] Postacchini F, Cinotti G, Perugia D, Gumina S (1993). The surgical treatment of central lumbar stenosis. Multiple laminotomy compared with total laminectomy. J Bone Joint Surg Br.

[29] Kalbarczyk A, Lukes A, Seiler RW (1998). Surgical treatment of lumbar spinal stenosis in the elderly. Acta Neurochir (Wien).

[30] Nerland US, Jakola AS, Solheim O, Weber C, Rao V, Lønne G (2015). Minimally invasive decompression versus open laminectomy for central stenosis of the lumbar spine: pragmatic comparative effectiveness study. BMJ.

[31] Jayarao M, Chin LS (2010). Results after lumbar decompression with and without discectomy: comparison of the transspinous and conventional approaches. Neurosurgery.

[32] Pao JL, Chen WC, Chen PQ (2009). Clinical outcomes of microendoscopic decompressive laminotomy for degenerative lumbar spinal stenosis. Eur Spine J.

[33] Mobbs RJ, Li J, Sivabalan P, Raley D, Rao PJ (2014). Outcomes after decompressive laminectomy for lumbar spinal stenosis: comparison between minimally invasive unilateral laminectomy for bilateral decompression and open laminectomy: clinical article. J Neurosurg Spine.

[34] Ho YH, Tu YK, Hsiao CK, Chang CH (2015). Outcomes after minimally invasive lumbar decompression: a biomechanical comparison of unilateral and bilateral laminotomies. BMC Musculoskelet Disord.

[35] Ozer AF, Oktenoglu T, Egemen E, Sasani M, Yilmaz A, Erbulut DU (2017). Lumbar single-level dynamic stabilization with semi-rigid and full dynamic systems: a retrospective clinical and radiological analysis of 71 patients. Clin Orthop Surg.

[36] Cha JR, Kim YC, Yoon WK, Lee WG, Kim TH, Oh JK (2017). The recovery of damaged paraspinal muscles by posterior surgical treatment for patients with lumbar degenerative diseases and its clinical consequence. J Back Musculoskelet Rehabil.

[37] Lonstein JE, Denis F, Perra JH, Pinto MR, Smith MD, Winter RB (1999). Complications associated with pedicle screws. J Bone Joint Surg Am.

[38] Papavero L, Thiel M, Fritzsche E, Kunze C, Westphal M, Kothe R (2009). Lumbar spinal stenosis: prognostic factors for bilateral microsurgical decompression using a unilateral approach. Neurosurgery.

[39] den Boogert HF, Keers JC, Marinus Oterdoom DL, Kuijlen JM (2015). Bilateral versus unilateral interlaminar approach for bilateral decompression in patients with single-level degenerative lumbar spinal stenosis: a multicenter retrospective study of 175 patients on postoperative pain, functional disability, and patient satisfaction. J Neurosurg Spine.

[40] Overdevest GM, Jacobs W, Vleggeert-Lankamp C, Thomé C, Gunzburg R, Peul W. Effectiveness of posterior decompression techniques compared with conventional laminectomy for lumbar stenosis. Cochrane Database Syst Rev. 2015(3):CD010036. 10.1002/14651858.CD010036.pub2. PMC11627146 25760812

